# Evaluation of baseline fracture risk in younger postmenopausal women with breast cancer using different risk assessment methods

**DOI:** 10.1007/s00256-020-03378-z

**Published:** 2020-01-24

**Authors:** Dagmar Schaffler-Schaden, Christoph Kneidinger, Gregor Schweighofer-Zwink, Maria Flamm, Bernhard Iglseder, Christian Pirich

**Affiliations:** 1 Institute of General Practice, Family Medicine and Preventive Medicine, Paracelsus Medical University, Strubergasse 21, 5020 Salzburg, Austria; 2 University Clinic for Nuclear Medicine and Endocrinology, Salzburg, Austria; 3 University Clinic for Geriatrics, Salzburg, Austria

**Keywords:** Breast cancer, Fracture risk, FRAX score, Postmenopausal, Trabecular bone score

## Abstract

**Objective:**

Controversy exists about the impact of bone mineral density (BMD) and fracture risk in newly diagnosed patients with breast cancer (BC). It is presumed that there are differences in BMD between women with BC and healthy controls. BMD is therefore considered as a potential marker to predict BC risk. This study was conducted to investigate the association of BMD, trabecular bone score (TBS) and fracture risk in younger postmenopausal women with hormone responsive BC.

**Methods:**

Overall, 343 women were examined. Women with BC were matched to a control group of the general population. Forty-nine women and fifty-nine controls were included in the final analysis. All subjects underwent dual energy x-ray absorptiometry (DXA) of the lumbar spine, femoral neck, and the total hip to evaluate bone mineral density. The 10-year fracture risk for a major osteoporotic fracture was assessed using the FRAX-score and the TBS-adjusted FRAX-Score, respectively.

**Results:**

Lumbar and femoral neck BMD were similar in BC patients and controls. No difference was found for TBS of the spine (1.38 ± 0.1 vs.1.36 ± 0.09) in the BC and the control group, respectively (*p* = 0.19). The 10- year probability for a major osteoporotic fracture (MoF) or femoral neck (FN) fracture was 6.1 (± 2.6%) and 0.9 (± 1.2%) in the BC group vs. 6.7 (± 3.5%) (*p* = 0.33) and 0.9 (± 1.1%) (*p* = 0.73) in the control group.

**Conclusion:**

Postmenopausal women younger than 60 years with breast cancer do not show any differences in baseline BMD, TBS, or TBS adjusted FRAX in comparison to controls.

## Introduction

The association of bone mineral density, fracture risk, and breast cancer is still unclear. Elevated bone mineral density (BMD) has been suggested a potential predictive marker for hormone responsive breast cancer as it reflects a woman’s lifetime exposure to estrogen [1]. Several studies indicated that women with a lower BMD have a lower risk for BC [2, 3]. Estrogen levels play a critical role in osteoporosis and are considered a risk factor for several cancers, particularly for breast cancer [4]. Osteoporosis commonly occurs in postmenopausal women with declining estrogen levels, but this risk is significantly increased by breast cancer treatment with aromatase inhibitors (AI), chemotherapy, radiation therapy, or treatment-related premature ovarian failure [5]. As AIs are established in adjuvant treatment for hormone receptor positive breast cancer in postmenopausal women, baseline and periodically BMD assessment with dual energy x-ray absorptiometry (DXA) for women undergoing AI therapy is recommended [6]. Although DXA is still the standard examination for osteoporosis diagnosis, studies reported that most individuals suffering incident fractures have a BMD above the commonly used therapeutic threshold T-score of -2.5 [7]. Hence, in recent years, additional parameters have been introduced to improve fracture risk prediction. The Fracture Risk Assessment algorithm (FRAX) was implemented in 2008 and summarizes several risk factors to estimate the 10-year probability for a hip or major osteoporotic fracture (hip, spine, forearm, or shoulder) [8]. The risk factors covered by FRAX are body mass index, current smoking, daily intake of three or more units of alcohol, previous fractures, parental hip fracture, use of corticosteroids, rheumatoid arthritis, or other causes for secondary osteoporosis. In addition, the Trabecular Bone Score (TBS) was recently introduced to assess bone quality [9, 10]. TBS is obtained from lumbar spine DXA as an index to evaluate bone microarchitecture and enhances the accuracy of fracture risk assessment. TBS was identified as a predictor of fracture risk independently from BMD, and, furthermore, TBS in combination with FRAX (TBS-adjusted FRAX) can be used to refine fracture risk prediction of the FRAX tool [11, 12].

The objective of this study was to investigate whether there is a difference in baseline BMD and 10-year fracture risk in younger postmenopausal women under 60 years with hormone responsive BC compared to a healthy control group using the TBS, the FRAX and the TBS- adjusted FRAX tool as three different risk assessment methods. Studies examining younger women are rare because breast cancer usually occurs at an advanced age, and routine osteoporosis screening is mostly recommended in women 65 years or older [13]. It is presumed that women with hormone receptor positive BC have a higher BMD and therefore have a lower fracture risk compared to an age-matched sample.

## Methods

This is a cross-sectional study. Data of the study population were collected retrospectively from electronic medical records. The study population (BC group) was compared to a randomly selected, age-matched control group (CG) of the general population. All women were examined in a single center and came from a geographically similar area. Standardized bone assessment was performed in all participants as described below. Overall, only women aged 50–59 years were included. Individuals with a BMI < 15 kg/m^2^ or > 30 kg/m^2^ were excluded due to exact fracture risk calculation using TBSiNsight© software. Furthermore, women receiving specific antiosteoporotic pharmacologic treatment (bisphosphonates, teriparatide, raloxifene, denosumab, zoledronic acid, or other) were excluded. Classification of osteopenia (−2.5 ≤ T-score < −1.0) and osteoporosis (T- score < −2.5) was performed according to WHO criteria.

The study population encompassed 343 postmenopausal women aged 50–59 years with confirmed hormone receptor (ER positive) positive breast cancer within the first 3 months after starting AI therapy, who are routinely referred to our clinic for baseline bone mineral density examination. Data of the study population were retrieved from the electronic medical records of the hospital from the years 2007 to 2013. Purchase of a special software was required for TBS assessment of women included before February 2011. Finally, 49 patients met the inclusion criteria. The main reasons for exclusion were age and a BMI <15 kg/m^2^ or > 30 kg/m^2^, most women with BC excluded were older than 59 years. Patients receiving AI therapy for more than 3 months were excluded as well as those with missing data. Data of the 59 women in the control group was randomly retrieved from the Paracelsus 10.000 study database of the years 2014–2015. This population-based study encompasses male and female participants aged 40–69 years from a given geographic area in Austria who are invited randomly on the basis of their residence. The study focuses on cardiovascular and metabolic diseases in the general population. Beside other examinations, healthy men and women aged 50–59 years were invited to a bone density scan (DXA) which was performed in our clinic. Overall, data of 1703 participants of the Paracelsus study was retrieved from the electronic medical records. Only female participants aged 50–59 years were included. Women with an anamnestic or documented history of breast cancer were excluded. Again, women with a BMI <15 kg/m^2^ or > 30 kg/m^2^ and specific antiosteoporotic pharmacologic treatment were excluded. Main exclusion criteria in the control group were age and gender as the primary sample included male participants as well. Relevant risk factors of all study participants were recorded at the beginning of the study. All women of both groups received a questionnaire containing relevant information for the FRAX calculation before DXA. This questionnaire included information about: age at first (menarche) and last menstruation (menopause), diabetes mellitus, chronic hepatic, renal or bowel disease, hyperthyroidism, rheumatic diseases, breast cancer or any other malignant tumors, ischemic stroke, stomach surgery, asthma, thrombotic events, previous fractures or parental hip fracture, alcohol and smoking habits, current medical therapy, and current back pain.

## Analysis of BMD, FRAX and TBS

All women underwent DXA of the lumbar spine, femoral neck, and total hip using Hologic Discovery QDR 4500, Bedford, MA, USA. TBS score was extracted from lumbar spine DXA and compared between both groups. TBS was evaluated using TBSiNsight© software, version 3.0.2.0., Med-Imaps, Bordeaux, France. According to McCloskey et al. [14], TBS values were categorized as degraded (TBS < 1.23), partially degraded (1.23 ≤ TBS ≤ 1.31), and normal bone (TBS > 1.31). T-scores of the hip, femoral neck, and lumbar spine were recorded and compared in both groups. The 10-year fracture risk was calculated using the country-specific FRAX tool provided by the WHO collaborating centre for Metabolic Bone Diseases, University of Sheffield, UK (https://www.sheffield.ac.uk/FRAX/tool.aspx?country=16).

## Statistics

Data management was performed using SPSS Version 8.0.120640 (IBM). The threshold for statistical significance was considered at *p* < 0.05. The independent t-test (two-tailed) was used to detect significant differences between the study and the control group. The Shapiro-Wilk test was used to check for normal distribution. Homogeneity of variance was determined using Levene’s test.

This study is registered in the International Clinical Trials Registry Platform (ICTRP, ID number: DRKS00016907). Ethical approval was obtained from the institutional Ethics Committee (ID number 415-E/2196/2–2017). All women gave informed consent.

## Results

The characteristics of the two groups are reported in Table 1, 2 and 3. 57.1% of women in the BC group had osteopenia (vs.42.4% in the control group), whereas 16.3% had osteoporosis (vs. 18.6% in the control group). Overall, 47.2% of women had osteopenia and 17.6% had osteoporosis. Women in the BC group had a slightly higher BMI, but the difference between the groups did not reach statistical difference (*p* = 0.058). There was a trend for higher T-scores of total hip, lumbar spine, and proximal femur in the control group although the difference was also not significant. Overall, the majority of women had a TBS considered normal >1.31 (71.3%).

**Table 1:** Demographic characteristics of women

	BC (*n* = 49)	CG (*n* = 59)	p
Age	55.6 ± 2.7	55.4 ± 2.3.	0.63
Weight	67.33 ± 7.97	64.89 ± 7.68	0.10
Height	163.63 ± 5.29	164.53 ± 6.86	0.45
BMI	25.18 ± 3.02	24.04 ± 3.14	0.05

BC = study population, CG: control group

**Table 2:** Anamnestic details of women

	BC	CG
Parental hip fracture	2	3
Atraumatic fracture	2	5
Smoking >20/d	2	5
Secondary osteoporosis	1	4

BC = study population, CG: control group

**Table 3:** Results

	BC	CG	p
T score hip	−0.66 ± 0.84	−0.63 ± 0.83	0.8
T score FN	−1.22 ± 0.85.	−1.14 ± 0.91	0.6
T score spine	−1.18 ± 1	−1.0 ± 1.22	0.4
TBS	1.38 ± 0.1	1.36 ± 0.09	0.1
FRAX MoF	6.4 ± 2.3%	6.7 ± 3.1%.	0.6
FRAX FN	1.1 ± 1.2%	1.1 ± 1.3%.	0.8
TBS adjusted FRAX MoF	6.1 ± 2.6%	6.7 ± 3.5%	0.3
TBS adjusted FRAX FN	0.9 ± 1.2%.	0.9 ± 1.1%	0.7

MoF: major osteoporotic fracture, FN: femoral neck

Seven women had a TBS value below 1.23, who were all in the BC group (7 vs. 0, *p* = 0.003). Thirty-eight women in the CG had a TBS considered as normal compared to 39 women in the BC group (*p* = 0.082).

The probability of a major osteoporotic fracture within 10 years based on the FRAX tool was 6.4 ± 2.3% for the BC group and 6.7 ± 3.1% for the control group, respectively. There was no difference between the two groups in terms of probability of MoF or FN fracture. Although significantly more women had a degraded TBS in the BC group, fracture risk in both groups was comparable. Using the TBS adjusted FRAX did not demonstrate a difference between the groups for MoF or FN (see Table 3).

## Discussion

The aim of this study was to compare baseline BMD, TBS, and fracture risk in women under the age of 60 with BC to a control group. Additionally to the common BMD measurements of the spine, proximal femur, and total hip we used the TBS, the FRAX and the TBS adjusted FRAX score to optimize fracture risk prediction as recently shown in a population of women with breast cancer receiving AI therapy [15]. According to WHO criteria, 57.1% of women in our BC group had osteopenia (42.3% of the control group), whereas 16.3% had osteoporosis (18.6% control group). Although our results were not significant, we observed a trend for the BC group having lower T-scores and a lower TBS, which is rather contradictory to the hypothesis that women with BC have a higher BMD than healthy women. A recent systematic review of the literature including 19 studies did not find a conclusive answer to the question whether an increased BMD is a significant risk factor for breast cancer [16]. In another sample of 2137 perimenopausal women, no significant difference in BMD was found between women having breast cancer and the non-BC group. There was even a trend in the BC group for a lower BMD in the femoral neck.This is consistent with our results [17]. As the majority of breast malignancies are hormone responsive, the treatment with AIs as antiestrogen agents is well established in postmenopausal women [18, 19]. However, regarding fracture risk of women with BC, study results are conflicting. A number of authors indicated that women with a higher BMD are at higher risk for developing breast cancer suggesting that there is a difference in baseline BMD of women with BC and healthy women [2, 3, 20–23]. Zambetti et al. revealed BMD as a significant prognostic factor for local or distant breast cancer recurrence. This study included patients regardless of BMD sites (lumbar spine, wrist, total hip, femoral neck, greater trochanter) although many women did not have a DXA of all five sites [2]. Other authors, in turn, did not distinguish women by menopausal status [21] or suggested an association of BMD and BC only for certain BMD sites. The strongest relationship has been found for lumbar spine BMD and BC. This effect was attributed to the impact of estrogen on trabecular bone [24, 25]. Brozek et al. reported that BMD was not found to be a predictor for BC in their study population of younger postmenopausal women [26]. In another large sample of 13.698 patients, BMD in general was identified as a weak predictor of breast cancer. Lumbar spine BMD in particular was not associated with an increased risk of BC, whereas age was identified as a strong-risk factor for breast cancer, with the mean age of women in this study being 67 years [27]. Since 25% of newly detected breast cancer cases concern women aged 75 or older, most studies exploring the association between BC and BMD included older postmenopausal women, only a few investigated younger women [16, 17, 28]. As Zhang et al. addressed in their recently published study regarding the bone–muscle unit, not only BMD but also muscle mass and -density are important factors associated with fracture risk. Since sarcopenia is usually rather a matter in the elderly population, we did not assess muscle mass and density in our patients [29]. Due to exact fracture risk assessment with TBSiNsight© software, we excluded obese patients since interference between BMI and TBS is a well-known confounding effect. A recent study concluded that TBS can also serve as a good marker for bone architecture in postmenopausal-obese women [30]. Overall, comparison of study results is hampered by heterogeneous study populations and differences in sites of BMD measurement. Hence, there is no evidence to date that BMD can be used as a predictive factor for breast cancer, and, therefore, BMD screening for women with increased BC risk is not yet recommended. This study has several limitations. The main limitation is the small number of study participants enrolled. The reason for this was that the study population included only women younger than 60 and breast cancer patients usually that are older. As people with no osteoporosis history have possibly a lower interest to participate in a study, baseline data of bone assessment in the control group could be biased. Information bias is possible as socially undesirable habits like heavy drinking or smoking are not reported correctly in the questionnaires. Since the women of the study group have already taken AI for 3 months, a (minimal) influence on BMD cannot be completely excluded. The main strength of the study is that it provides additional information about the fracture risk of younger postmenopausal women with the TBS adjusted FRAX tool and the comparison with an age-matched, population-based control group. Comparability of data is enhanced, as all women were examined in a single institution and underwent the same standardized assessment methods.

In conclusion, our study did not confirm previous study results indicating an association between BMD and breast cancer. Women in the study group did not differ in BMD or fracture risk compared to a healthy control group.
